# Photochemically induced dynamic nuclear polarization NMR on photosystem II: donor cofactor observed in entire plant

**DOI:** 10.1038/s41598-018-36074-z

**Published:** 2018-12-14

**Authors:** Geertje J. Janssen, Pavlo Bielytskyi, Denis G. Artiukhin, Johannes Neugebauer, Huub J. M. de Groot, Jörg Matysik, A. Alia

**Affiliations:** 1 University of Leiden, Leiden Institute of Chemistry, Einsteinweg 55, P.O. Box 9502, 2300 RA Leiden, The Netherlands; 2 Universität Leipzig, Institute of Analytical Chemistry, Johannisallee 29, D-04103 Leipzig, Germany; 3 Westfälische Wilhelms-Universität Münster, Organisch-Chemisches Institut and Center for Multiscale Theory and Computation, Corrensstraße 40, D-48149 Münster, Germany; 4 Universität Leipzig, Institute of Medical Physics and Biophysics, Härtelstr. 16-18, D-04107 Leipzig, Germany

## Abstract

The solid-state photo-CIDNP (photochemically induced dynamic nuclear polarization) effect allows for increase of signal and sensitivity in magic-angle spinning (MAS) NMR experiments. The effect occurs in photosynthetic reaction centers (RC) proteins upon illumination and induction of cyclic electron transfer. Here we show that the strength of the effect allows for observation of the cofactors forming the spin-correlated radical pair (SCRP) in isolated proteins, in natural photosynthetic membranes as well as in entire plants. To this end, we measured entire selectively ^13^C isotope enriched duckweed plants (*Spirodela oligorrhiza*) directly in the MAS rotor. Comparison of ^13^C photo-CIDNP MAS NMR spectra of photosystem II (PS2) obtained from different levels of RC isolation, from entire plant to isolated RC complex, demonstrates the intactness of the photochemical machinery upon isolation. The SCRP in PS2 is structurally and functionally very similar in duckweed and spinach (*Spinacia oleracea*). The analysis of the photo-CIDNP MAS NMR spectra reveals a monomeric Chl *a* donor. There is an experimental evidence for matrix involvement, most likely due to the axial donor histidine, in the formation of the SCRP. Data do not suggest a chemical modification of C-13^1^ carbonyl position of the donor cofactor.

## Introduction

The photochemically induced dynamic nuclear polarization (photo-CIDNP) magic-angle spinning (MAS) NMR technique is a unique analytical tool to extract detailed information at the atomic level from photochemically active photosynthetic reaction centers (RCs) as well as from another protein undergoing light-induced electron transfer^1,2^. The solid-state photo-CIDNP effect allows for strong signal enhancement by light-induced induction of non-Boltzmann nuclear spin polarization. The photo-CIDNP appears to be an intrinsic property of natural RCs occurring in frozen isolated RC samples as well as in liquid membranes. The spin-chemical origin of the effect is in the meanwhile understood, in which up to three mechanisms, called three-spin mixing (TSM), differential decay (DD) and differential relaxation (DR) run in parallel. The phenomenon has been predicted to occur at Earth’s magnetic field and recently revised in terms of level crossings and anti-crossings^3^. While the observed chemical shift refers to the electronic ground state obtained after the photocycle, photo-CIDNP intensities are related in a non-trivial manner to the local electron spin density in the spin-correlated radical pair (SCRP). As an analytical tool^1^, photo-CIDNP MAS NMR has been applied to various RCs and the strong increase in sensitivity and selectivity by the solid-state photo-CIDNP effect in combination with selective isotope labeling allowed for direct observation of the primary radical pair in entire cells of selectively ^13^C labeled purple bacteria and cyanobacteria *Synechocystis*. Here we show that even larger biological structures, *i*.*e*., entire plants as complete duckweed plants including roots, can be studied inside a MAS rotor by NMR.

**Figure 1:**
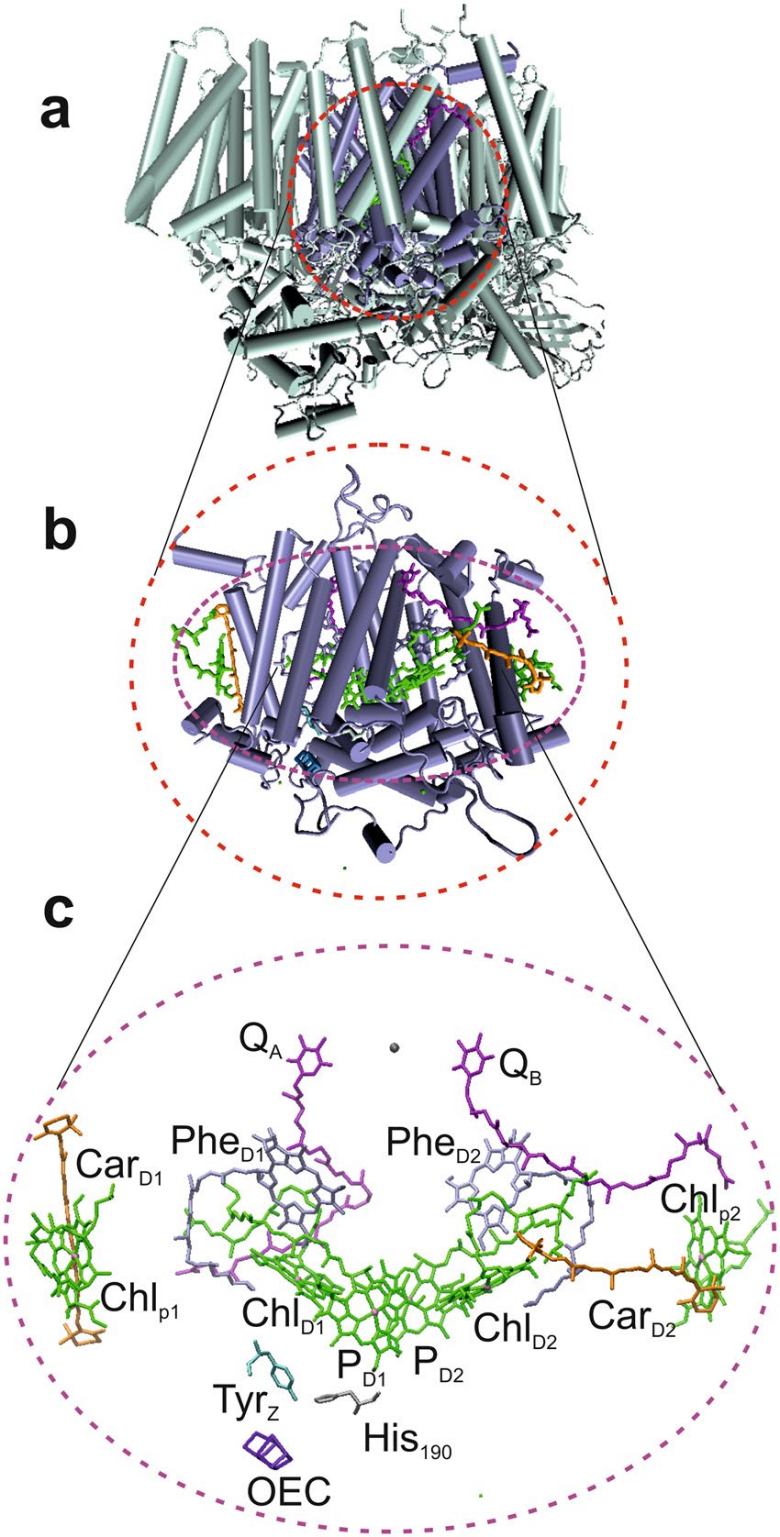
The PS2 core complex (**a**) is embedded inside the thylakoid membrane (not shown). At the heart of the core complex, the PS2 reaction center (RC) is found (**b**) and mainly formed by the central D1 and D2 polypeptides. The PS2 RC contains several cofactors (**c**): two central Chls (P_D1_ and P_D2_), two accessory Chls (Chl_D1_ and Chl_D2_), two pheophytins (Phe_D1_ and Phe_D2_), two quinones (Q_A_ and Q_B_), and two peripheral Chls (Chl_P1_ and Chl_P2_). These cofactors are arranged in two symmetrical branches, an active D1 branch (left) and an inactive D2 branch (right). Two β-carotenoids (Car_D2_ and Car_D1_) are associated with the PS2 core complex. At the P_D1_ side, a tyrosine residue Tyr_Z_ is in between P_D1_ and the oxygen evolving system (OEC).

The photosynthetic machinery in plants converts photon energy from sunlight into chemical energy by oxidizing water and reducing carbon dioxide while releasing molecular oxygen as a side-product. To achieve this, two large trans-membrane protein complexes, photosystem 2 (PS2) and photosystem 1 (PS1), operate in series (for a review, see Blankenship^4^). In plant cells, both PS2 and PS1 are located in the thylakoid membrane in the chloroplasts, pumping protons from the lumen to the stroma site. For structural and functional studies on PS2, it is possible to selectively remove PS1 from the membrane to obtain PS2 enriched membranes, so-called BBY preparations (Berthold, Babcock and Yocum)^5^. From these membranes, the PS2 core particles (Fig. 1a) can be isolated by removing peripheral light-harvesting complex 2 (LHC2) using Triton X as detergent as described by van Leeuwen *et al*.^6^. Further removal of the light-harvesting core antenna proteins CP43 and CP47 leads to the PS2 RC or D1D2 complex (Fig. 1b) comprising the D1 and D2 polypeptides in which the two branches of cofactors are symmetrically arranged (Fig. 1c). The cofactors consist of two inner chlorophyll *a* (Chl *a*) molecules (P_D1_ and P_D2_), two accessory chlorophylls (Chl *a*_D1_ and Chl *a*_D2_), two pheophytins (Phe *a*) and two quinones (Q) arranged in two symmetric branches. In addition, the PS2 RC contains two β-carotenes, two peripheral chlorophylls (Chl *a*_p1_ and Chl *a*_p2_) and the water splitting manganese cluster, also known as the oxygen-evolving complex (OEC). PS2 has the highest oxidation power known in living nature (+1.2 V), allowing to drive the water splitting. On the other hand, PS1 has the strongest reductive power known in living nature, which is required for the CO_2_ reduction. How PS2 is able to produce and maintain such oxidative strength within a protein environment and without changing the HOMO-LUMO gap (680 nm) is, despite extensive research by optical-kinetic and magnetic resonance techniques and the availability of high-resolution (1.9 Å) x-ray data, not yet fully understood.

Until now, photo-CIDNP MAS NMR studies on plant PS2 have been restricted to experiments on isolated D1D2 RC preparations from spinach (*Spinacia oleracea*) due to the difficulty to incorporate selective isotope labels into plant RCs. Based on the data obtained from natural abundance D1D2, a first assignment of the light-induced signals was made and the donor was identified to be a single Chl *a* cofactor^7,8^. The intensity pattern provided strong evidence for a pronounced asymmetry of the electronic spin-density distribution within the monomeric Chl *a* donor assigned to P_D1_. While free Chl *a* in solution has the highest electron-spin density being located at pyrrole ring II^9^, the Chl *a* donor inside the D1D2 complex shows a shift of electron-spin density towards rings III and IV^7,8,10^. A possible explanation of the electron-spin density shift was suggested to be the presence of a local electrostatic field close to ring III, created for example by the protonation of the keto-group of ring V^7^. Since it appears that the electron-spin density on the oxidized donor is also localized on the axial histidine, a tilting of the axial histidine towards pyrrole ring IV causing π-π overlap of both aromatic systems was proposed. In this “hinge model”, the charge state of the histidine suggested a negatively charged Chl *a*-histidine complex becoming a neutral radical in the photo-oxidized state^8,10^ (for review, see Najdanova *et al*.^11^). Such electronic structures might allow for the remarkable increase in redox potential of PS2 in comparison to bacterial RCs.

To study photosynthetic units larger than D1D2, such as core preparations, BBY membranes or entire plants, selective isotope labeling is required. In this work, we present the first photo-CIDNP MAS NMR data obtained at various levels of PS2 selectively ^13^C labeled in the Chl *a* and Phe *a* cofactors. The label incorporation succeeded in the aquatic plant duckweed (*Spirodella oligorrhiza*), which has been chosen since it has shown previously to successfully incorporate ^15^N as well as ^2^H-, ^13^C- or ^17^O-labeled tyrosine^12^.

## Results and Discussion

### Photo-CIDNP MAS NMR spectra obtained from 13C-labeled BBY, thylakoids and plants

**Figure 2:**
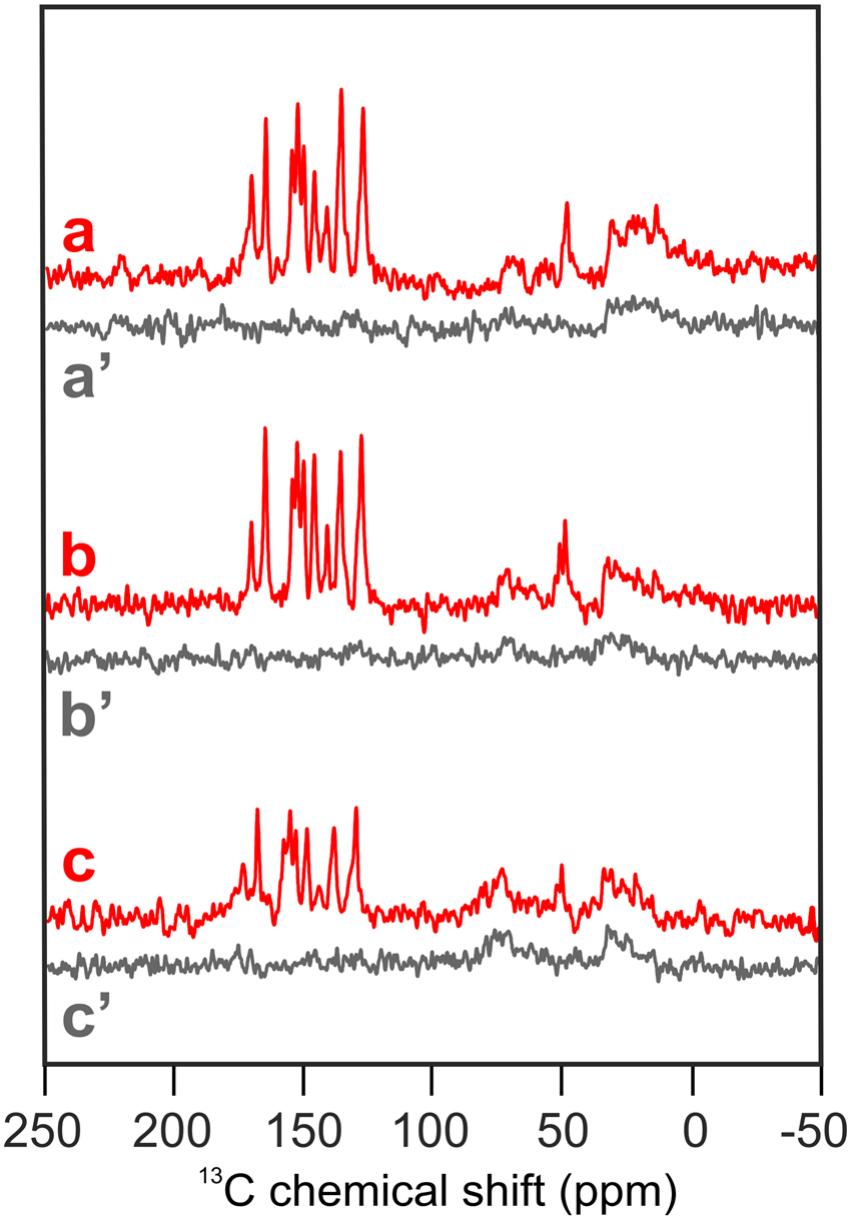
^13^CMAS NMR spectra of selectively 4-ALA ^13^C-isotope labeled BBY preparation (**a**), thylakoid membranes (**b**) and entire plants (**c**) of the aquatic plant *Spirodela oligorrhiza* obtained under continuous illumination (red). Spectra (**a’**–**c’**) (grey) show the corresponding spectra obtained under dark conditions. All spectra were obtained at a magnetic field of 4.7 T and a temperature of 235 K with a MAS frequency of 8 kHz and a cycle delay of 4 s.

In Fig. 2, Spectra a-c, shown in red, are obtained under continuous illumination of selectively 4-ALA ^13^C-isotope labelled BBY (a), thylakoid membranes (b) and from entire plants (c) of the duckweed *Spirodella oligorrhiza*. The same Figure shows in black Spectra a’ to c’ obtained from the same samples in the dark. The dark spectra show only weak and broad positive signals in the aliphatic region between 0 and 50 ppm and, due to the C-α of the amino acids of the protein backbone, between 60 and 80 ppm.

**Figure 3:**
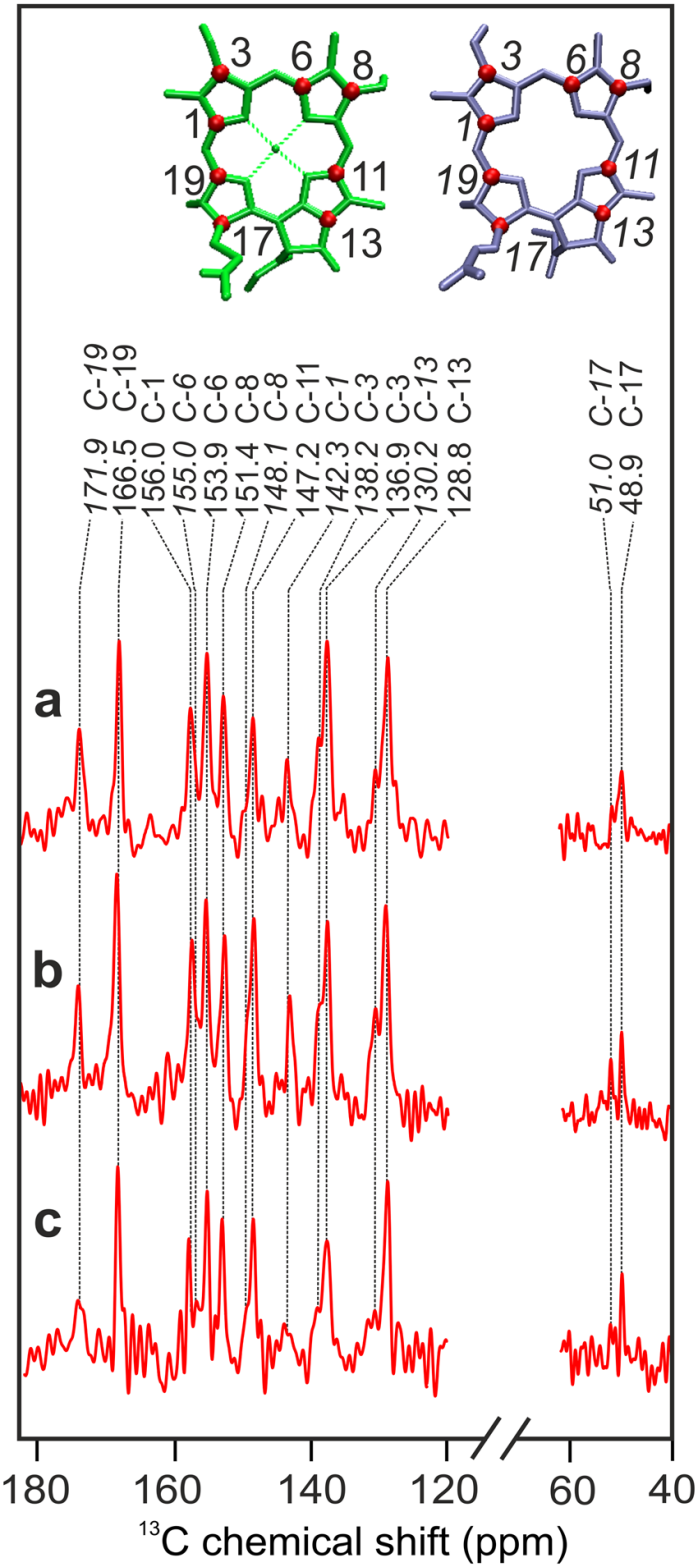
Detailed views of the aromatic and aliphatic regions of the ^13^C photo-CIDNP MAS NMR spectra (**a**–**c**) depicted in Fig. 2. The position of the ^13^C-isotope labeled carbons in the Chl *a* donor (green) and the Phe *a* acceptor (purple) are visualized by red dots (top). Assigned centerbands are visualized by dashed lines (Table 1). Signals assigned to the Phe *a* acceptor are denoted in Italics. The numbering is according to the IUPAC nomenclature.

**Figure 4:**
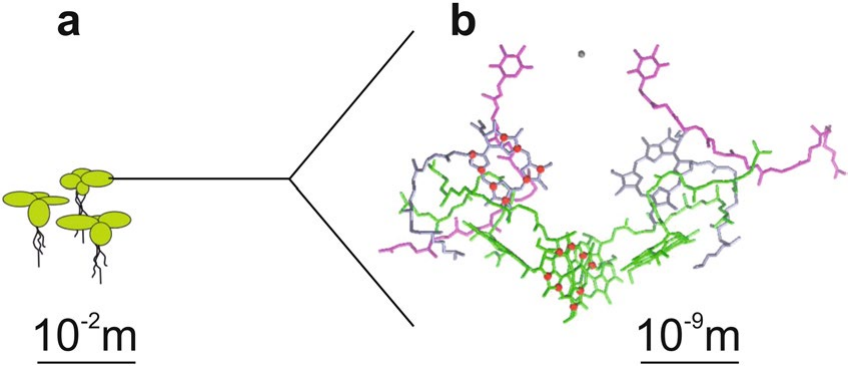
By combining specific ^13^C isotope labelling with photo-CIDNP MAS NMR, in the plant of *Spirodela oligorrhiza* (duckweed, (**a**), the red labelled nuclei of the active Chl *a* and Phe *a* cofactors (red dots) of the PS2 RC (**b**) are directly detected without further isolation.

The 15 signals that are light-induced by the solid-state photo-CIDNP effect can be straightforwardly recognized. Figure 3 shows an expansion of the spectral region for the light-induced signals in Fig. 2. The data are presented with the tentative assignment (see below) to the labelled carbons in the Chl *a* donor and Phe *a* acceptor. For convenience, the isotope label patterns of the Chl *a* donor (green) and Phe *a* acceptor (purple, numbering in Italics) obtained by biosynthetic labeling with 4-ALA are indicated with numbering at the red dots on top of Fig. 3. The data show that:

(i) The solid-state photo-CIDNP effect allows to observe the two cofactors forming the SCRP directly from entire plants without any further isolation (Fig. 4). This implies that ^13^C photo-CIDNP MAS NMR is able to study selectively microscopic structures in the Ångström range within macroscopic units up to the dimension of centimeters. Thus, we propose that such “in-plant” photo-CIDNP MAS NMR could be considered as a new application in a growing field of in-cell solid-state NMR^13–15^.

(ii) The comparison of the ^13^C photo-CIDNP MAS NMR spectra of BBY particles (spectrum a), thylakoid membrane (spectrum b) and intact leaves (spectrum c) does not reveal any significant difference, neither in the chemical shift nor in the intensity. Minor changes in the relative intensity of the signals might occur at 51.0 and 147.2 ppm, although they are in the limits of the noise. Hence, both, the chemical shift values and the overall intensity pattern are highly conserved among spectra obtained from these three levels of isolation. The consistency observed with the highly sensitive NMR spectroscopy provides clear experimental evidence that the electronic states of the cofactors forming the SCRP are not affected by the preparation procedure. Thus, the data obtained from a D1D2 RC preparation^7,10^ indeed reflect the genuine natural state occurring in intact thylakoid membranes and plants.

(iii) The below discussed assignment of the light-induced signals corroborates the concept of a SCRP formed by a single donor Chl *a* and a single acceptor Phe *a*. While ^13^C photo-CIDNP MAS NMR intensities are correlated to local electron-spin densities^16^, in case of ^13^C labelling, the polarizations are equilibrated by spin-diffusion. On the other hand, the spread of intensity allows to observe nuclei which are otherwise difficult to detect^17^. Spin-diffusion allows for example for the detection of the nearby aliphatic signals for the two C-17 carbons from the donor and acceptor. While in unlabelled samples, all signals are conveniently assigned to a single Chl *a* cofactor, i.e. the donor^7^, upon 4-ALA ^13^C-labelling also acceptor signals are observed. Effects of selective ^13^C isotope labelling on the spin-dynamics have also been observed in heliobacterial RCs^18^ but are not yet theoretically permeated. The donor signals are in general stronger than the acceptor signals, suggesting an additional contribution of the DR mechanism^19^.

### No signals from PS1

It is remarkable that the light-induced spectra of BBY on the one hand, and of thylakoids and plants on the other hand are very similar, although thylakoid and plant samples contain the full photosynthetic machinery, including both PS2 and PS1, while BBY contains PS2 only. The absence of photochemically active PS1, which shows an entirely emissive light-induced ^13^C photo-CIDNP MAS NMR, can be due to several reasons: (i) The position of the quinones: while the quinones on PS2 are easily accessible and instantaneously reduced upon addition of sodium dithionite, the quinones in PS1 are not expected to be readily reduced upon direct freezing and measurement. To successfully reduce PS1, incubation at room temperature after addition of the reductant and exposure to light at room temperature and during freezing are necessary. (ii) The low pH of the sample environment (~pH 4.5): *in vivo*, the active site of PS1 is situated at the alkaline (stroma) side of the thylakoid membrane (pH 8), while PS2 functions at the acidic lumen side (pH 4) of the membrane. Acidic conditions strongly decrease PS1 stability and activity, while both donor and acceptors side of PS2 are known to remain intact under strong acidic conditions.

### Photo-CIDNP in PS2 preparations containing the OEC

To allow for the solid-state photo-CIDNP effect, a SCRP on P_D1_ and Phe *a* with sufficiently long lifetime, i.e., some 10 s of ns, is required. One might assume that such long lifetime cannot sustain in the presence of the OEC^20^. Our results demonstrate the occurrence of the same SCRP in the preparations of larger PS2 complexes (with the OEC present) with sufficiently long lifetime as in the D1D2 preparation (where the OEC is lost). There are, indeed, arguments for the interruption of the OEC activity in the present set of experiments: (i) The electron transfer from the OEC requires an intact hydrogen bonding network around Tyr_Z_, the intermediary electron carrier between the OEC and P_D1_ Chl *a*^21^. The rate of re-reduction of P_D1_^+^ decreases at low pH^22^. This effect has been suggested to be linked to a distortion or breakage of the hydrogen bond between TyrZ and the nearby D1-His190, which has an estimated p*K*_a_ of 4.5-5.3^23-25^ (for review see Styring *et al*.^26^). (ii) Below a pH of 5.5, the nanosecond kinetics of electron transfer quickly slows down to the microsecond. The pH dependence provides a natural mechanism for physiological regulation of electron transfer from the OEC, allowing for dissipation of excess excitation energy by the RC at high light conditions^27^. In all experiments shown here, after reduction with Na_2_S_2_O_3_ in the rotor, a pH below 5.0 has been reached in preparations of the larger PS2 complexes, i.e., in plants, BBY and core preparations. Furthermore, acidification of the lumen space down to a pH of 5.0 or slightly below occurs *in vivo* under strong light conditions^28^. Hence, the pH allows for lifetimes of the SCRP sufficiently long to induce the solid-state photo-CIDNP effect. (iii) The temperature of the experiment (~235 K) blocks the re-reduction from the OEC. The reaction cycle of the OEC^29^ is strongly inhibited at 230 K^30-32^. If we assume that the OEC remains blocked in its S2 state, even at pH 6–7.5, the re-reduction rate would slow down to 250 ns^33^. (iv) Also in core preparations, Q_B_ is lost while Q_A_ remains bound to the protein pocket^34^. In the preparations of larger PS2 complexes, both quinones are, at least initially, present. It is possible that Q_B_ is lost upon reduction prior to the measurement. In this case, after some photocycles, Q_B_ will be saturated and the light-induced electron transfer becomes cyclic. At high light intensities, also under natural conditions, photo-reduction of quinones has been shown to occur leading to double reduction of Q_A_ which finally can result in the release of Q_A_ as Q_A_H_2_ (up to 63% in 80 min)^35^. In core preparations, double reduction of Q_A_ can be significantly promoted by the addition of a strong reductant and subsequent illumination^36,37^. In BBY preparations, illumination under reductive conditions has been demonstrated to cause 100% double reduction of Q_A_^38^. Hence, the experimental conditions allow for observation of the SCRP of PS2 despite the OEC being present.

### ^13^C photo-CIDNP MAS NMR on isolated PS2 RCs from spinach and duckweed

**Figure 5:**
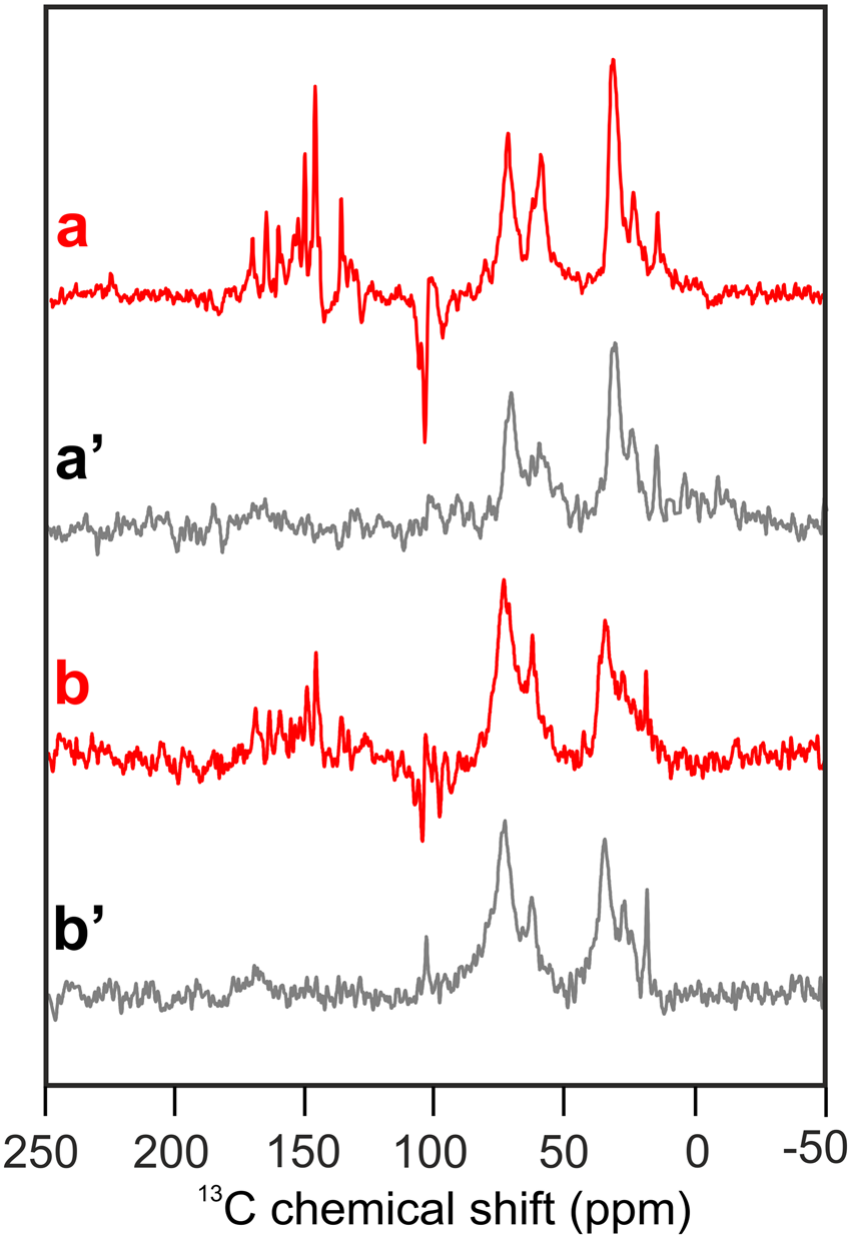
^13^C MAS NMR spectra of natural abundance D1D2 particles of spinach (**a**) and natural abundance core complexes of duckweed (**b**) obtained under continuous illumination (red). Spectra (a’ and b’) show the corresponding spectra obtained under dark conditions (grey). All spectra were obtained at a magnetic field of 4.7 T and a temperature of 235 K with a MAS frequency of 8 kHz and a cycle delay of 4 s.

As a next step, we will compare PS2 data from spinach and duckweed. The impossibility to obtain D1D2 RCs from duckweed forces us to compare Core particles to D1D2 RC preparations from spinach. In Fig. 5, shown in red, the ^13^C photo-CIDNP MAS NMR Spectra a and b are obtained under continuous illumination of a D1D2 preparation from unlabelled spinach (a) and of core particles from unlabelled duckweed (b). Spectra a’ and b’, depicted in grey, show the corresponding dark spectra. As expected, the dark spectra show signals in the aliphatic region between 0 and 50 ppm as well as a broad signal between 60 and 80 ppm. The dark signals are due to the C-α of the amino acids of the protein backbone and the (glycine) buffer used for sample preparation. In the photo-CIDNP MAS NMR spectra in Fig. 5, absorptive (positive) and emissive (negative) light-induced signals occur in the region between 80 and 170 ppm. Although the isolated RC shows stronger light-induced features than the Core complex, chemical shifts and intensity patterns between both light-induced spectra are very similar, proving that the PS2 from both organisms function in a similar fashion. The data provide strong evidence that both the electronic ground-state structure as well as the radical-pair structure of the photochemical machinery of PS2 remain unchanged when comparing the two different plant species and upon isolation of D1D2 from the Core complex. This is in line with an optical study using femtosecond transient absorption spectroscopy, which revealed a conservation of the efficient electron transfer rate constants upon isolation of the D1D2 complex from PS2 Core^39^. Also the mechanism of electron transfer, with Chl_D1_ acting as the primary electron donor and Phe_D1_ as the primary acceptor, was found to be the same in both systems^39^. Hence, our data demonstrate that the photochemical machinery of PS2 is robust against various states of isolation. The highest level of isolation, the D1D2 RC, is not disturbed, and essentially functioning in the same way as in full plants. Furthermore, we have shown that this machinery is very similar in PS2 of spinach and duckweed.

**Figure 6:**
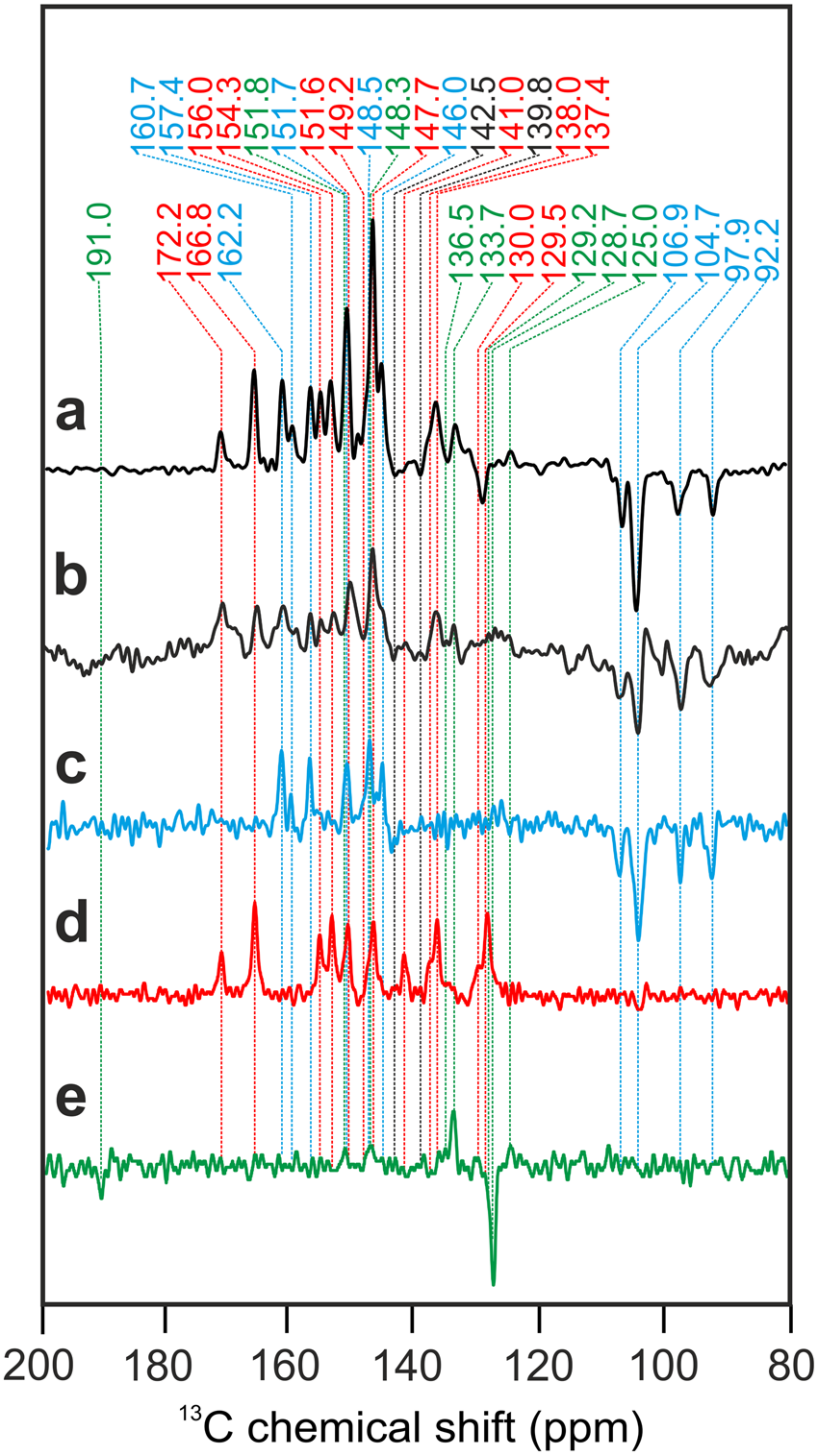
Enlarged view of the ^13^C photo-CIDNP MAS NMR spectra shown in Fig. 5 obtained from natural abundance D1D2 particles of spinach (**a**) and core complexes of duckweed (**b**). Furthermore, ^13^C photo-CIDNP MAS NMR spectra are shown obtained from 5-ALA (**c**, blue), 4-ALA (**d**, red) and 3-ALA (**e**, green) ^13^C-labelled thylakoid preparation. The color code of the dotted lines refers to the selective label patterns. All spectra were obtained at a magnetic field of 4.7 T and a temperature of 235 K with a MAS frequency of 8 kHz and a cycle delay of 4 s.

To compare the spectra of the D1D2 preparation of spinach and that of the Core preparation of duckweed in more details, Fig. 6 provides an enlarged view. Spectrum a, originating from the D1D2 preparation of unlabeled spinach, is reproducing well data from the literature^7,8^. Previously, 23 light-induced signals were observed, most of them were tentatively assigned to the aromatic ring carbons of the Chl *a* donor. Absence of signal doubling provided a hint for a monomeric donor. The emissive signals between 90 and 130 ppm were identified as the four methine carbons. It has been proposed that the broad emissive response between 140 and 145 ppm and the emissive signal at 129.2 ppm originate from the axial histidine of the Chl *a* donor^10^. Spectrum b originates from the Core preparation from unlabelled duckweed. Despite the overall great similarity of chemical shifts and other spectral features, the emissive signal between 129 and 130 ppm is apparently missing. Also, the broad emissive signals at 142.5 and 139.8 ppm might be extinguished. All these emissive features are assigned to the axial histidine^8^. MAS rotation might lead to orientation effects in membrane-based samples, changing the relative intensity contribution between π-systems having different orientations^10^. Therefore, the collective absence of these emissivive features in the membrane sample suggests a common origin and backs the assignment to the axial histidine.

### Assignment of ^13^C photo-CIDNP MAS NMR signals of 5-ALA, 4-ALA and 3-ALA labeled samples

As shown above, the possibility to introduce ^13^C isotope labels allows to observe the effect in larger systems. Furthermore, isotope labelling enables for a more detailed characterization of the SCRP and the identification of possible abnormalities. To this end, we will now aim for signal assignment by using selectively 5-ALA, 4-ALA and 3-ALA labeled samples. Since it allows for efficient label introduction, we prepared thylakoid samples from duckweed with various ^13^C isotope label patterns to study the structure of the SCRP of PS2. In Fig. 6, Spectra c to e have been obtained from samples were the Chl *a* and Phe *a* molecules were specifically ^13^C labeled according to the 5-ALA (c), 4-ALA (d) and 3-ALA (e) labeling patterns (for labeling patterns, see Supplementary Fig. S1). By using specifically labeled samples, it is possible to selectively highlight eight carbons in each active Chl *a* or Phe *a* cofactor and assign them by using literature values obtained from Chl *a* aggregates^40^ and plant Phe *a* reconstituted in bacterial RCs^41^. In Table 1, we aim to reconstruct the spectra of the unlabeled PS2 core and D1D2 samples (Fig. 6, Spectra a and b) by using the data obtained from selectively ^13^C-labeled thylakoid membranes (Fig. 6, Spectra c to e). Again, the consistency of many marker lines in labeled and unlabeled samples clearly demonstrates the conservation of the electronic structure of the PS2 Chl *a* donor upon isolation of core particles from thylakoid membranes. The detailed discussion on the assignment can be found in Supplementary Information. We can conclude from the assignments:

**Table 1:** Assignment of the light-induced signals observed in 3-, 4-, and 5-ALA-labelled thylakoid preparations of duckweed (Fig. 6) to carbon positions of either the Chl *a* donor or the Phe *a* acceptor cofactor.

Light-induced ^13^C signal (ppm)		Assignments	Comments
**191.0**	*E*,*w*	**190 C-13** ^1^ **Phe**	
(not observed)		190.6 C-13^1^ Chl	
**172.2**	*A*	**173 C-19 Phe**	
**166.8**	*A*	**170.0 C-19 Chl**	
**162.2**	*A*	**162.0 C-14 Chl**	
**160.7**	*A*	**161 C-16 Phe**	
**157.4**	*A*	**154.0 C-16 Chl**	
**156.0**	*A*	**155.9 C-1 Chl**, 156 C-6 Phe	
**154.3**	*A*	**154.4 C-6 Chl**	
**151.8**	*A*,*w*	(no match)	
**151.7**	*A*	**150.7 C-4 Chl**, 150 C-9 Phe, 151 C-14 Phe	
**151.6**	*A*	**147.2 C-11 Chl**	
**149.2**	*A*	**145 C-8 Phe**	
**148.5**	*A*	**150.7 C-4 Chl**, 150 C-9 Phe	
**148.3**	*A*,*w*	(no match)	
**147.7**	*A*	**146.2 C-8 Chl**	
**146.0**	*A*	**147.2 C-9 Chl**	
**142.5**	*E*	(no match)	Histidine
**141.0**	*A*	**142 C-1 Phe**	
**139.8**	*E*	(no match)	Histidine
**138.0**	*A*,*S*	**138 C-11 Phe**	
**137.4**	*A*	**138.0 C-3 Chl**, 136 C-3 Phe	
(not observed)		137 C-4 Phe	
**136.5**	*A*,*b*,*w*	**136.1 C-2 Chl**, 136 C-7 Phe	
**133.7**	*A*	133.4 C-7 Chl, **134.0 C-12 Chl**	
(not observed)		131 C-2 Phe	
**130.0**	*A*,*S*	**133 C-13 Phe**	
**129.5**	*A*	**126.2 C-13 Chl**	
**129.2**	*E*	**128 C-12 Phe**	Histidine?
**128.7**	*E*	129 C-3^1^ Phe, **128 C-12 Phe**	
**125.0**	*A*,*w*	**126.2 C-3** ^**1**^ **Chl**	
**107.7**	*A*,*w*	(no match)	
**106.9**	*E*	108.2 C-10 Chl, **107 C-15 Phe**	
**104.7**	*E*	102.8 C-15 Chl, **105 C-10 Phe**	
**97.9**	*E*	98.1 C-5 Chl, **97 C-5 Phe**	
**92.2**	*E*	93.3 C-20 Chl, **93 C-20 Phe**	
**51.0**	*A*	**52 C-17 Phe**	(see Fig. 3)
**48.9**	*A*	**51.4 C-17 Chl**	(see Fig. 3)
**29.3**	*A*,*w*	**32.5 C-17**^1^ **Chl**, 32 C-17^1^ Phe	(not shown)
**19.6**	*A*,*w*	**20.2 C-8**^**1**^ **Chl**, 20 C-8^1^ Phe	(not shown)

The reference chemical shifts of Chl *a* are obtained from solid aggregates of Chl *a*^40^ and of Phe *a* from isolated RCs of *R*. *sphaeroides* R26 carrying Phe *a* instead of BPhe *a*^41^. For details, see Supplementary Information.

Abbreviations: *A* = enhanced absorptive; *b* = broad; Chl = chlorophyll *a*; *E* = emissive; Phe = pheophytin *a*; *S* = shoulder; *w* = weak.

(1) While the aromatic system appears largely undisturbed, the absence of the donor C-13^1^ carbonyl carbon and the occurrence of the two weak, unassigned so far, absorptive signals at 151.8 and 148.3 ppm in the 3-ALA pattern teases for further studies.

(2) The emissive signals at 142.5 and 139.8 ppm, assigned to the axial histidine^10,11^, do indeed not originate from the Chl *a* and Phe *a* cofactors. The emissive signal at 129.2 ppm, however, might be caused by the C-12 carbon of the acceptor, although we cannot rule out that that acceptor signal is overlaying the histidine signal. Thus, the matrix is involved into the formation and evolution of the SCRP.

Therefore, below we will test possible chemical modifications of the donor by means of chemical-shift calculations. Possible chemicals modifications^7,8,42^ are:

(i) Chl *a* protonated at position 13^1^, positively charged, [Chl-OH]^+^;

(ii) Chl *a* protonated at position 13^1^, neutral, [Chl-OH];

(iii) a Schiff-base formation at the C-13^1^ of the Chl *a* donor, [Chl-NH_2_]^+^.

### Possible chemical modifications of the Chl a donor

The question remains about the missing donor C-13^1^ signal and the possible signals at 151.8 and 148.3 ppm. One might assume that the C-13^1^ signal has been significantly shifted by a chemical modification at the C-13^1^ position. This idea is attractive because it might explain the unchanged optical properties although the electro-chemical properties of these cofactors, esp. the extremely high redox potential, are highly unusual. Possible chemical modifications are protonation and Schiff-base formation at this carbonyl side (see above). To explore the possibility of such chemical modifications, theoretical calculations have been applied. The results of the calculations are listed in Table 2. It can be seen that the calculated shifts are in reasonable agreement with the experimental values for Chl *a*, with a root-mean-square deviation of 5.3 ppm and a maximum absolute deviation of 10.8 ppm. Changes in these shifts upon chemical modification are expected to be of higher accuracy due to the possibility of error cancellation. This is supported by the observation that these changes are much more consistent with the corresponding changes calculated with the BP86 functional (given in Supplementary Table S1) than the actual Chl *a* chemical shifts.

**Table 2:** Calculated chemical shifts of substituted derivatives of Chl *a*:.

Carbon atom number	Exp. Chl *a*	[Chl *a*]	[Chl-OH]^+^	[Chl-OH]	[Chl-NH_2_]^+^
**13** ^1^	190.6	179.8	174.0 (−5.8)	161.9 (−17.9)	157.7 (−22.1)
**19**	170.0	162.9	172.5 (9.6)	166.2 (3.3)	169.7 (6.8)
**14**	162.0	156.1	161.1 (5.0)	156.0 (−0.1)	158.5 (2.4)
**1**	155.9	146.4	155.7 (9.3)	151.4 (5.0)	153.7 (7.3)
**6**	154.4	146.1	156.6 (10.5)	148.7 (2.6)	154.2 (8.1)
**16**	154.0	157.4	164.4 (7.0)	159.5 (2.1)	161.0 (3.6)
**4**	150.7	142.2	151.7 (9.5)	145.4 (3.2)	149.6 (7.4)
**11**	147.2	146.7	149.6 (2.9)	146.0 (−0.7)	148.0 (1.3)
**9**	147.2	143.5	150.0 (6.5)	144.1 (0.6)	148.2 (4.7)
**8**	146.2	141.9	147.9 (6.0)	140.8 (−1.1)	147.3 (5.4)
**3**	137.0	134.1	139.0 (4.9)	134.4 (0.3)	138.6 (4.5)
**2**	136.1	131.2	136.3 (5.1)	130.9 (−0.3)	135.7 (4.5)
**12**	134.0	134.5	127.4 (−7.1)	129.3 (−5.2)	129.3 (−5.2)
**7**	133.4	132.7	137.7 (5.0)	130.0 (−2.7)	137.2 (4.5)
**13**	126.2	129.9	122.2 (−7.7)	130.8 (0.9)	121.3 (−8.6)
**3** ^**1**^	126.2	128.4	125.5 (−2.9)	127.4 (1.0)	126.0 (−2.4)
**10**	108.2	101.6	104.4 (2.8)	104.3 (2.7)	103.9 (2.3)
**15**	102.8	104.7	101.1 (−3.6)	114.2 (9.5)	98.6 (6.1)
**5**	98.1	96.3	99.7 (3.4)	98.2 (1.9)	99.4 (3.1)
**20**	93.3	91.9	95.9 (4.0)	92.5 (0.6)	95.1 (3.2)
**17**	51.4	55.5	55.6 (0.1)	53.4 (−2.1)	56.0 (0.5)
**17** ^1^	32.5	38.7	42.3 (3.6)	39.6 (0.9)	41.6 (2.9)
**8** ^1^	20.2	24.7	24.1 (−0.6)	23.6 (−1.1)	24.3 (−0.4)

[Chl *a*] – calculated Chl *a*.

[Chl-OH]^+^ – Chl *a* protonated at position C-13^1^, positively charged.

[Chl-OH] – Chl *a* protonated at position C-13^1^, neutral.

[Chl-NH_2_]^+^ – Chl *a* as a Schiff base at position C-13^1^, positively charged.

The carbon atom numbers are colored according to the labelled pattern: 3, 4 and 5-ALA.

All calculations were carried out with KT2/TZP. The difference between the chemical shifts of modified and unmodified Chl *a* is presented in parentheses.

In all three calculated structures with a chemical modification, the ppm value of carbon C-13^1^ lowered. The lowest value, around 160 ppm, is found for the Schiff-base. Therefore, from a spectroscopic view, we would not rule out an assignment of the two possible signals around 150 ppm. However, in the structure^43^, there is no amino acid around the donor Chl *a* able to form a Schiff base. The two calculated structures with protonated C-13^1^ positions are not very likely from the chemical shift values. Hence, a chemical modification as explanation of the absence of the signals of C-13^1^ from the donor is unlikely.

## Conclusions

(1) The solid-state photo-CIDNP effect can be observed in entire plants. The observation of photo-CIDNP by ^13^C MAS NMR directly in plants allows application for exploration of SCRP in systems in which a further isolation has not yet been established. (2) The active photochemical machinery forming the SCRPs of PS2 is conceptually the same as for bacterial RCs, provided the hole transfer on the donor side is blocked by lowering the pH. (3) This machinery remains essentially unaffected upon isolation from plant level to the D1D2 preparation. (4) PS2 RCs in spinach and duckweed show essentially identical ^13^C photo-CIDNP MAS NMR spectra demonstrating structural and functional conservation between these plant species. (5) The SCRP is formed by a Chl *a* donor-Phe *a* acceptor pair. In both cofactors, the ^13^C chemical shifts of the aromatic π-system are close to standard conditions. (6) Based on the comparison of selectively labeled PS2 with natural abundance PS2, involvement of the protein matrix in the formation of SCRP has been demonstrated. It appears that signals not originating from the cofactor can be straightforwardly assigned to a histidine. (7) It does not appear likely that the donor cofactor is chemically modified at the C-13^1^ position. Therefore, to explain the unusual properties of the donor, we assume that conformational effects, esp. related to the axial histidine^10^, electrostatic fields^7^ and dielectric properties of the protein^44^ act together.

## Methods

### Strains and culture conditions

*Spirodella oligorrhiza* was grown under aseptic conditions on half-strength Hunter’s medium under continuous light (20 μEm^−2^s^−1^) at 25 °C. The medium was continuously bubbled with sterile air containing 5% CO_2_. For selective ^13^C labeling, fully grown plants were exposed to δ-aminolevulinic acid (ALA, purchased from Cambridge Isotope Laboratories), isotopically ^13^C labeled at carbon position 3 (3-ALA), 4 (4-ALA) or 5 (5-ALA) to a final concentration of 1.4 mM in half-strength Hunter’s medium at pH 4.8. After 7 days plants were harvested and used directly for sample preparation or frozen in liquid nitrogen and stored at −80 °C until use.

### Determination of the ^13^C-label incorporation

Chl *a* was extracted from plants grown in ^13^C-ALA supplemented half-strength Hunter’s medium (labeled sample) and from unlabeled plants (reference sample), according to the following procedure: Plants were homogenized in half-strength Hunter’s medium and centrifuged for 10 min at 16,000 × *g*. The supernatant was removed and the residue was dissolved in 1 mL MeOH. The methanolic solution was centrifuged for 5 min at 300 × *g*. The green supernatant was separated from the blue and white residue and dried under a gentle stream of N_2_. The sample was re-suspended in acetone, loaded on a cellulose column and pure Chl *a* fractions were eluted with petroleum ether/acetone (7/3 *v/v*). The solvent was evaporated under N_2_ flow and the pure Chl *a* was stored at −20 °C under a dry nitrogen atmosphere in the dark.

### Liquid chromatography-mass spectrometry (LC-MS)

Mass spectra were measured with a LTQ–FT hybrid mass spectrometer (Thermo Fisher, Waltham, MA, USA). Spectra were measured in ESI mode, with a source temperature of 200 °C, source voltage of 3.8 kV and tube lens voltage 150 V. Chl *a* was dissolved in 90% EtOH and 10% 10 mM ammonium acetate to a final concentration of ~1 mg/mL. The sample was infused with a flow rate of 10 μL min^−1^. The biosynthetic route from ALA to Chl *a* and Phe *a* is described in Schulten *et al*.^45^. Two molecules of ALA are asymmetrically condensed to form the pyrrole porphobilinogen. Four molecules of porphobilinogen tetramerize, and prior to macrocycle ring closure, the last pyrrole ring is inverted via a spiro-intermediate. Upon incorporation of 3-^13^C, 4-^13^C or 5-^13^C-ALA, a maximum of 8 ^13^C-atoms can be incorporated into each Chl *a* or Phe *a* molecule, resulting into the specific labeling patterns shown in Supplementary Fig. S1. Based on the LC-MS spectra observed in the region of m/z = 893.5 ([M]^•+^; C_55_H_72_O_5_N_4_Mg) to *m/z* + 8 (maximum ^13^C incorporation) the total level of incorporation (P_tot_) was determined via an iterative procedure as described earlier by Schulten *et al*.^45^. Since ALA is a precursor of both Chl *a* and Phe *a*, it is assumed that the level of label incorporation into Phe *a* and Chl *a* is identical. Supplementary Fig. S2 shows a typical mass spectrum of Chl *a* isolated from *S*. *oligorrhiza* grown under standard conditions (Fig. S2, A) and in the presence of the ^13^C lableled ALA precursor (Fig. S2, B). An average of 75% ^13^C isotope enrichment is accomplished.

### Preparation of D1D2 and Core complex

The natural abundance samples of isolated D1D2 from spinach were prepared as described earlier in Matysik *et al*.^7^. The PS2 core complexes from duckweed were isolated according to the procedures described by van Leeuwen *et al*.^6^.

### Preparation of ^13^C labeled BBY

Selectively ^13^C labeled BBY membranes were isolated according to the procedure as described by Berthold *et al*.^5^ that was adjusted for micro scale preparation using 10 g of 4-ALA labeled duckweed plants as a starting material. After solubilization in MES buffer (20 mM MES, 15 mM NaCl_2_, 5 mM MgCl_2_, pH 6.0) starch was removed by 5 minutes slow centrifugation (Sorvall SS34) at 80 × *g*. The Chl *a* concentration of the reaction mixture was determined using a Moran Assay and adjusted to 1 mg/mL. Triton-100 was added to a final concentration of 5% (w/v). After incubation on ice under constant stirring for 20 minutes the sample was centrifuged for 20 minutes at 25,000 × *g*. The pellet was resuspended in MES buffer to remove Triton-100 and again centrifuged for 20 minutes at 25,000 × g. The product (1.5 mL of 1.7 mg/mL Chl *a*) was stored at −80 °C in BTS-200 buffer (20 mM tricine, 10 mM MgCl_2_, 5 mM CaCl_2_, 10 mM MgSO_4_, 0.2 M sucrose and 0.03% (w/v) *n*-dodecyl-β-D-maltoside, pH 6.5).

### NMR sample preparation

D1D2 was directly loaded into an optically transparent 4-mm NMR sapphire rotor. All other samples were previously reduced by the addition of sodium dithionite to a final concentration of 100 mM under oxygen-free atmosphere and low-light conditions and loaded into sapphire rotors. For experiments on BBY, 200 µL of BBY product (Chl *a* concentration of 1.7 mg/mL) was washed twice with sucrose free BTS-200 buffer (BTS-0) and resuspended in 70 µL of BTS-0 before reduction. For preparation of thylakoid samples, ~200 mg isotope labeled plants were homogenized in a minimal amount of half-strength Hunter’s medium at pH 5.8 under near dark conditions. Starch was removed with slow centrifugation at 60 × *g* for 3 minutes using an Eppendorf 5415D. The supernatant was collected and centrifuged for 10 min at 16000 × *g*, and the pellet was resuspended in half strength Hunter’s medium. For experiments on entire plants ~100 mg plants were incubated in a solution containing 100 mM sodium dithionite for 10 minutes under nitrogen atmosphere in complete darkness. Plants were carefully stacked inside an optically transparent 4-mm NMR sapphire rotor, and half-strength Hunter’s medium was added to fill the rotor. In all cases, the filled rotor was directly loaded into the NMR probe and cooled under slow spinning (800 Hz) to 235 K. Membrane and plant samples were always freshly prepared and directly frozen inside the NMR apparatus. The estimated concentration of PS2 RC present at different levels of purification is shown in Supplementary Table S2.

### Photo-CIDNP MAS NMR experiments

^13^C-MAS NMR experiments were performed on a DMX-200 NMR spectrometer (Bruker Biospin GmbH, Karlsruhe, Germany). All spectra have been obtained at a sample temperature of 235 K and with a spinning frequency of 8 kHz. The data were collected with a spin echo pulse sequence under two-pulse phase modulation carbon-proton decoupling^46^. Optimized ^1^H and ^13^C 90° pulse lengths were 5.1 and 3.1 μs, respectively. The cycle delay of 4 s was used, the acquisition time was 35 ms. About 7 k and 20 k scans were used to record continuous illumination spectra of ^13^C-labeled and natural abundance samples, respectively. Between 3.5 k and 10 k scans were used to record the respective dark spectra. The ^13^C NMR spectra were referenced to the COOH response of solid L-tyrosine hydrochloride at 172.1 ppm. Photo-CIDNP MAS NMR spectra have been obtained under continuous illumination with a 1000-Watt xenon arc lamp^47^.

### Spectral fitting

The fitting of the light induced signals obtained by photo-CIDNP MAS NMR has been performed using IgorPro version 6.01 (Lake Oswego, Oregon).

### Computational details

Structure optimizations of the compounds [Chl *a*], [Chl-OH]^+^, [Chl-OH], [Chl-NH_2_]^+^ were carried out with the program package ADF^48^ employing the exchange–correlation functional KT2^49^ and the triple-zeta TZP basis set^50^. As a consistency check, also calculations with the BP86 functional^51,52^ have been performed. The corresponding results are given in the Supporting Information (Table S1). Dispersion interactions were taken into account using the D3 correction by Grimme *et al*.^53,54^ with Becke–Johnson damping^55–57^. The numerical quality for the density fit and grid construction procedures were set to “good”. Tight convergence criteria were applied during the SCF cycles (1.0e^−8^ a.u. for the norm of the Fock and density matrices) as well as in geometry optimization (1.0e^−4^ and 1.0e^−4^ a.u. for changes in energy and gradient, respectively). The optimized geometries were verified as minima on the potential energy surfaces with normal mode analyses. The same computational settings were used for the generation of potentials required for calculations of ^13^C nuclear magnetic shieldings with the NMR module^58–62^. Chemical shifts were calculated with respect to the tetramethylsilane (TMS) shieldings obtained with the same settings as described above.

## Electronic supplementary material

Supplementary Information
