# Supplementary material for: Photochemically induced dynamic nuclear polarization NMR on photosystem II: donor cofactor observed in entire plant
Source: Sci Rep. 2018 Dec 14;8:17853. doi: 10.1038/s41598-018-36074-z (PMC6294776; doi:10.1038/s41598-018-36074-z)
Supplement: Supplementary file 1 — Supplementary Information [file 41598_2018_36074_MOESM1_ESM.pdf]

SUPPLEMENTARY INFORMATION FOR

Photochemically induced dynamic nuclear polarization NMR on photosystem II: donor cofactor observed in entire plant

Geertje J. Janssen,[1] Pavlo Bielytskyi,[2] Denis G. Artiukhin,[3] Johannes Neugebauer,[3] Huub J.M. de Groot,[1] Jörg Matysik,[2] A. Alia[1,4]**

[1]University of Leiden, Leiden Institute of Chemistry, Einsteinweg 55, P.O. Box 9502, 2300 RA Leiden, The Netherlands;

[2]Universität Leipzig, Institute of Analytical Chemistry, Johannisallee 29, D-04103 Leipzig, Germany;

[3]Westfälische Wilhelms-Universität Münster, Organisch-Chemisches Institut and Center for Multiscale Theory and Computation, Correnssstraße 40, D-48149 Münster, Germany;

[4]Universität Leipzig, Institute of Medical Physics and Biophysics, Härtelstr. 16-18, D-04107 Leipzig, Germany

Assignment of ^{13}C photo-CIDNP MAS NMR signals of 5-ALA, 4-ALA and 3-ALA labeled samples

In Spectrum c (Fig. 6, main text), depicted in blue, obtained from 5-ALA ^{13}C-labeled thylakoid samples, 10 light-induced signals can be distinguished, four of them are emissive and are straightforwardly assigned to the methane-bridge carbons. The tree most deshielded signals (162.2, 160.7 and 157.4 ppm) are enhanced absorptive and well resolved, and can be assigned to carbons C-14 (Chl *a*), C-16 (Phe *a*) and C-16 (Chl *a*). Also the assignment of the signal at 146.0 ppm to C-9 (Chl *a*) can be done directly. The two signals at 151.7 and 148.5 ppm might originate either from the donor or the acceptor. Since the well assigned signals originating from Chl *a* have similar intensity, it is reasonable to also assign the signals at 151.7 and 148.5 ppm to the donor. Such an assignment implies that signals from the acceptor are weaker as also observed for the signal at 160.7 ppm. The fact that the donor signals are stronger enhanced than the acceptor signals is probably due to an additional contribution by the DR mechanism selectively acting on the donor[1]. The four emissive signals around 100 ppm show shoulders and satellites. Assuming that the DR adds positive intensity to the donor signals, we assign the four strongly emissive signals to the acceptor, implying that the shoulders and satellites originate from the donor. The assignment based on selective isotope labeling supports the idea of a monomeric donor and a monomeric acceptor forming the radical pair. All ^{13}C labeled positions have been assigned to observed intensities, except for C-4 of the acceptor which resonates at 137 ppm[2] in RCs of *Rhodobacter sphaeroides*. The measured chemical shifts deviate from those of monomeric Chl and Phe in solution by less than 3.4 ppm, for the C-16 of Chl *a*, which suggests that the cofactor positions are mildly disturbed. Remarkable is the weak emissive feature at 142.5 ppm which might be caused by the unlabeled axial histidine.

In Spectrum d of Figure 6, presented in red color, 12 signals are observed, all are enhanced absorptive, originating from the 4-ALA label pattern (see Fig. 3, top). Furthermore, two enhanced absorptive aliphatic signals reported on Fig. 3 at 51.0 and 48.9 ppm could be conveniently assigned to the carbons C-17 of Phe *a* and Chl *a*, respectively. It appears that also in this label pattern two distinguished intensity levels are observed due to equilibration by spin-diffusion, allowing to assign the stronger signals to the Chl *a* donor and the weaker signals to the Phe *a* acceptor. The most deshielded signals, occurring at 172.2 and 166.8 ppm, can be straightforwardly assigned to the Carbons C-19 of Phe *a* and Chl *a*, respectively. These two isolated signals nicely present the intensity ratio of about 1:2 in favor of the donor. The next three signals (156.0, 154.3, 151.6 ppm) can be conveniently assigned to the donor carbons C-1, C-6 and C-11, respectively, assuming that the signal of C-6 of the acceptor is hidden between the signals at 156.0 and 154.3 ppm. Next, the strong signal at 147.7 ppm can be assigned to C-8 of the donor, implying that its shoulder at 149.2 ppm originates from the C-8 of the acceptor. The weaker signal at 141.0 ppm belongs to C-1 of the acceptor. The signal at 137.4 with shoulder at 138.0 ppm matches well to the C-11 of Phe *a* and C-3 of Chl *a*. One might assume that a weak acceptor signal from C-3 is hidden under this feature. Finally, the signal at 129.5 ppm with a shoulder at 130.0 ppm certainly originates from C-13 of Chl *a* and Phe *a*, respectively[3]. In addition, the 4-ALA label pattern produces signals at 51.0 and 48.9 ppm (Fig. 3), which allows to complete a consistent assignment of all signals expected in a 4-ALA sample.

The light-induced shifts also in the 4-ALA sample appear only modestly disturbed (maximum 4.2 ppm for C-8 of Phe *a*), as compared to the standard chemical shifts. Trials to base the assignment on two-dimensional spectroscopy failed, probably due to limited ^{13}C label incorporation. The assignment obtained here cannot rule out that dramatic chemical shift changes occur. However, we can safely state that for the 5- and 4-ALA label patterns, which together provide an almost complete aromatic system, a rather straightforward and consistent

assignment is possible implying that the aromatic rings of both donor and acceptor are almost undisturbed. This implies that the remarkable electronic properties of PS2 are induced by effects beyond the aromatic system. In fact, a previous photo-CIDNP MAS NMR study on the Special pair donor of the bacterial RC of *Rhodobacter sphaeroides* has shown that the tuning effect there is due to the side-chains[4].

To explore the direct proximity of the aromatic system, the 3-ALA labeled sample was studied. In the 3-ALA pattern, only two carbons, C-2 and C-12, participate in the aromatic ring structure, carry significant electron spin density and are expected to appear with significant intensity[5]. Furthermore, one would expect that neighboring labeled positions, i.e. C-3^1 and C-13^1, will gain some intensity via spin-diffusion from these positions[6,7]. The chance to observe the carbons C-17^1, C-18 C-7 and C-8^1 appears to be rather low. Hence, one would expect a spectrum showing four strong and eight weaker signals. One also would expect, relying on the 5- and 4-ALA assignments (see above), that the donor signals are in particular strong[8,9]. Obviously, the experimentally obtained spectrum (Fig. 6, spectrum e) looks rather different and, lacking a complete set of signals, a consistent assignment will be difficult.

The strongest enhanced absorptive signal appears at 133.7 ppm, and can be straightforwardly assigned to C-12 of the donor which is expected at 133.4 ppm[3]. Close to it, around 136.5 ppm, a weak and broad positive feature is present and can be assigned to C-2 of the donor that is expected to occur at 136.1 ppm. In any case, we have now identified the two main sources of polarization on the donor: all appear with enhanced absorptive intensity. Now, we want to search for the two main sources on the acceptor: C-2 of Phe *a*, expected at 131 ppm, is missing. The C-12 of Phe *a*, expected at 128 ppm, matches well to the strongly emissive signal at 128.7 ppm. Hence, it appears that the acceptor signals, showing both enhanced absorptive and emissive intensity, are close to a sign-change. This phenomenon has also been

observed in ^{13}C-labelled heliobacterial RCs[10]. In that case, spin-diffusion on the molecule might be hampered since contribution from both signs will cancel each other.

Now, we will search for signals of the positions C-3^1 and C-13^1 of the donor that are expected to gain sensitivity directly from C-2 and C-12. Carbon C-3^1 of Chl *a*, expected at 126.2 ppm, is observed at 125.0 ppm. However, there is no evidence for the occurrence of the carbonyl carbon C-13^1 (expected at 190.6 ppm) from the donor. On the other hand, several positions that were expected to be very weak can be located in the aliphatic range (not shown). Thus, it is possible to observe C-8^1 (expected at 20.2 and observed at 19.6 ppm) and also a weak absorptive signal that can be assigned to C-17^1 (expected at 32.5 and observed at 29.3 ppm). These two signals would also match to the acceptor (C-17^1 is expected at 32.5 and C-8^1 at 20 ppm). However, the occurrence of even the C-17^1 suggests an assignment to the donor since strong positive intensity spread by spin-diffusion is required. It is remarkable, that carbonyl carbon C-13^1 from the donor is missing, although even C-8^1 and C-17^1 are observable. Searching signals from the positions C-3^1 and C-13^1 of the acceptor, we assume that C-3^1 (expected at 129 ppm) is hidden under the emissive intensity at 128.7 ppm, and the carbonyl carbon C-13^1 (expected at 190 ppm) is a thin emissive line at 191 ppm. Since this carbonyl signal is emissive, it must originate from a neighbor of the strongly emissive signal at 128.7 ppm, which has been assigned to C-12 of the acceptor. Hence, for both donor and acceptor, the two "pumping positions" C-2 and C-12 as well as the two "receiving positions" C-3^1 and C-13^1 provide a reasonable pattern, except for the remarkable absence of the C-13^1 carbonyl carbon of the donor.

Puzzling are also the two weak absorptive features at 151.8 and 148.3 ppm, since for them, there is no candidate to assign, neither from Chl *a* nor from Phe *a*. These signals are about 15 ppm distant from all other expected frequencies and, thus, these weak signals would suggest a significant change of the ground-state electronic structure in the periphery of the aromatic ring. Their absorptive intensity suggests that these signals occur from the donor.

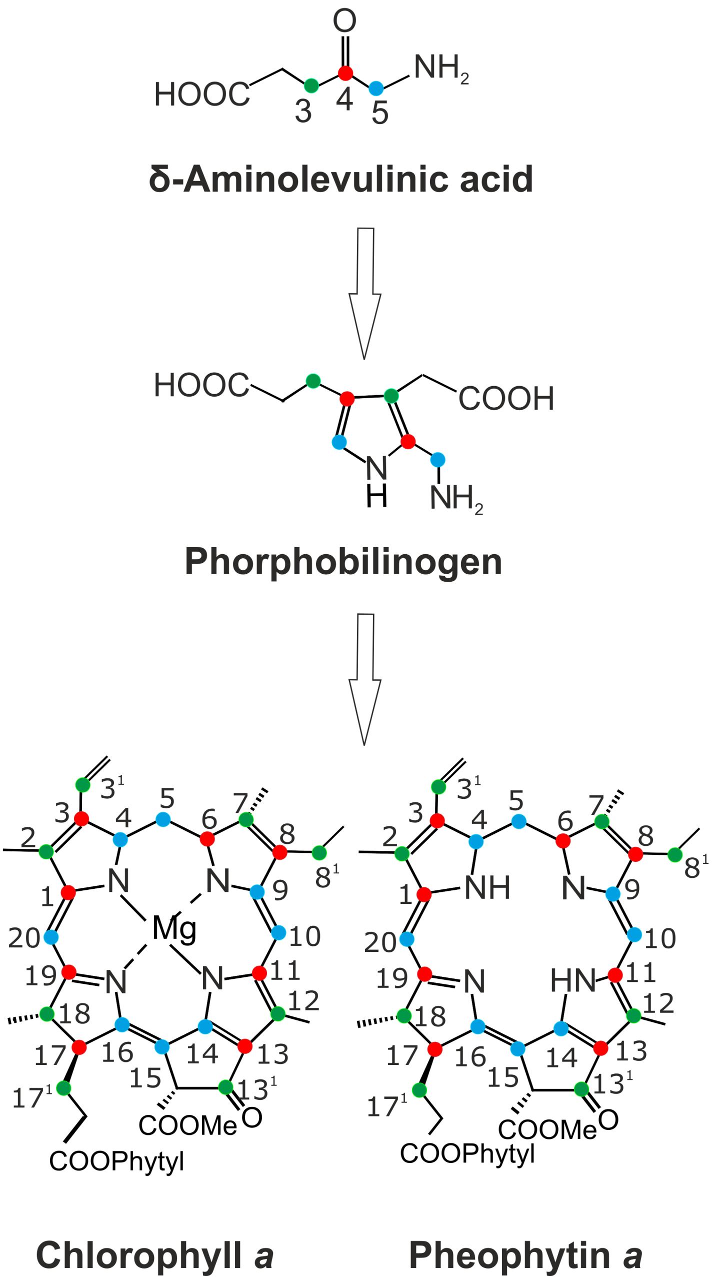

Figure S1. Incorporation of selective ^{13}C labels into Chl *a* and Phe *a* cofactors by feeding of 3-aminolevulinic acid (ALA), 4-ALA or 5-ALA precursors to growing plants. Depending on the position of the ^{13}C label within the precursor (at either positions 3, 4 or 5), the Chl *a* and Phe *a* molecules are labelled during the natural biosynthesis pathway at the positions visualized by the colored dots. Accordingly, the cofactors obtain 3-ALA (green), 4-ALA (red) or 5-ALA (blue) labelling patterns.

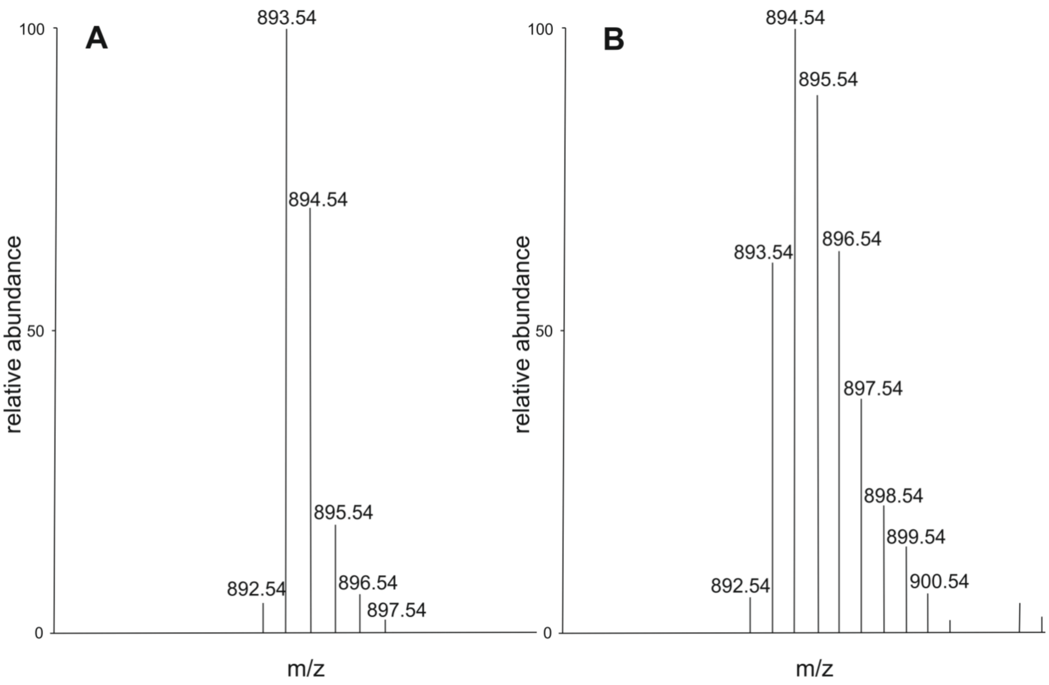

Figure S2. Mass spectra obtained by LC-MS measurements of Chl *a* isolated from *S. oligorrhiza* leaves grown under standard conditions (A) and in the presence of the ^{13}C 4-ALA precursor (B).

Tab. S1 Comparison of computational functional for calculating the chemical shifts of substituted derivatives of Chl *a*:

Carbon atom number	Exp. Chl *a*	[Chl *a*]		[Chl-OH]$^+$		[Chl-OH]		[Chl-NH_2]$^+$	
		BP86	KT2	BP86	KT2	BP86	KT2	BP86	KT2
13^1	190.6	191.0	179.8	183.8<br>(-7.2)	174.0<br>(-5.8)	169.4<br>(-21.6)	161.9<br>(-17.9)	167.0<br>(-24)	157.7<br>(-22.1)
19	170.0	171.5	162.9	181.6<br>(10.1)	172.5<br>(9.6)	174.8<br>(3.3)	166.2<br>(3.3)	178.7<br>(7.2)	169.7<br>(6.8)
14	162.0	164.6	156.1	170.2<br>(5.6)	161.1<br>(5.0)	164.5<br>(-0.1)	156.0<br>(-0.1)	167.2<br>(2.6)	158.5<br>(2.4)
1	155.9	156.0	146.4	165.5<br>(9.5)	155.7<br>(9.3)	160.9<br>(4.9)	151.4<br>(5.0)	163.5<br>(7.5)	153.7<br>(7.3)
6	154.4	155.0	146.1	166.6<br>(11.6)	156.6<br>(10.5)	157.3<br>(2.3)	148.7<br>(2.6)	163.6<br>(8.6)	154.2<br>(8.1)
16	154.0	165.5	157.4	174.2<br>(8.7)	164.4<br>(7.0)	168.2<br>(2.7)	159.5<br>(2.1)	170.9<br>(5.4)	161.0<br>(3.6)
4	150.7	151.3	142.2	161.2<br>(9.9)	151.7<br>(9.5)	154.3<br>(3.0)	145.4<br>(3.2)	159.1<br>(7.8)	149.6<br>(7.4)
11	147.2	155.5	146.7	157.8<br>(2.3)	149.6<br>(2.9)	154.2<br>(-1.3)	146.0<br>(-0.7)	156.3<br>(0.8)	148.0<br>(1.3)
9	147.2	152.0	143.5	158.8<br>(6.8)	150.0<br>(6.5)	152.3<br>(0.3)	144.1<br>(0.6)	157.0<br>(5.0)	148.2<br>(4.7)
8	146.2	150.7	141.9	156.9<br>(6.2)	147.9<br>(6.0)	149.4<br>(-1.3)	140.8<br>(-1.1)	156.3<br>(5.6)	147.3<br>(5.4)
3	137.0	144.7	134.1	150.0<br>(5.3)	139.0<br>(4.9)	145.0<br>(0.3)	134.4<br>(0.3)	149.5<br>(4.8)	138.6<br>(4.5)
2	136.1	140.8	131.2	146.3<br>(5.5)	136.3<br>(5.1)	140.4<br>(-0.4)	130.9<br>(-0.3)	145.7<br>(4.9)	135.7<br>(4.5)
12	134.0	144.2	134.5	137.0<br>(-7.2)	127.4<br>(-7.1)	137.8<br>(-6.4)	129.3<br>(-5.2)	138.6<br>(-5.6)	129.3<br>(-5.2)
7	133.4	141.9	132.7	147.1<br>(5.2)	137.7<br>(5.0)	139.1<br>(-2.8)	130.0<br>(-2.7)	146.6<br>(4.7)	137.2<br>(4.5)
13	126.2	138.6	129.9	130.9<br>(-7.7)	122.2<br>(-7.7)	139.7<br>(1.1)	130.8<br>(0.9)	130.0<br>(-8.6)	121.3<br>(-8.6)
3^1	126.2	136.9	128.4	133.8<br>(-3.1)	125.5<br>(-2.9)	136.1<br>(-0.8)	127.4<br>(1.0)	134.3<br>(-2.6)	126.0<br>(-2.4)
10	108.2	108.9	101.6	111.6<br>(2.7)	104.4<br>(2.8)	111.5<br>(2.6)	104.3<br>(2.7)	111.1<br>(2.2)	103.9<br>(2.3)
15	102.8	112.9	104.7	109.8<br>(-3.1)	101.1<br>(-3.6)	122.8<br>(9.9)	114.2<br>(9.5)	107.3<br>(5.6)	98.6<br>(6.1)
5	98.1	104.2	96.3	107.8<br>(3.6)	99.7<br>(3.4)	106.2<br>(2.0)	98.2<br>(1.9)	107.5<br>(3.3)	99.4<br>(3.1)

**20**	93.3	99.5	91.9	103.8 (4.3)	95.9 (4.0)	99.9 (0.4)	92.5 (0.6)	103.0 (3.5)	95.1 (3.2)
**17**	51.4	57.2	55.5	57.5 (0.3)	55.6 (0.1)	54.3 (-2.9)	53.4 (-2.1)	57.9 (0.7)	56.0 (0.5)
**17^1**	32.5	38.2	38.7	38.5 (0.3)	42.3 (3.6)	39.3 (1.1)	39.6 (0.9)	38.1 (-0.1)	41.6 (2.9)
**8^1**	20.2	24.9	24.7	24.4 (-0.5)	24.1 (-0.6)	23.8 (-1.1)	23.6 (-1.1)	24.6 (-0.3)	24.3 (-0.4)

[Chl *a*] - calculated Chl *a*
$[Chl\text{-}OH]^+$ - Chl *a* protonated at position C-13^1, positively charged
[Chl-OH] - Chl *a* protonated at position C-13^1, neutral
$[Chl\text{-}NH_2]^+$ - Chl *a* as a Schiff base at position C-13^1, positively charged
The carbon atom numbers are colored according to the labeled pattern: 3, 4 and 5-ALA
The difference between the chemical shifts of modified and unmodified Chl *a* within one calculation method is presented in parentheses.

Tab. S2 Comparison of concentration of PS2 RC in different samples used for photo-CIDNP studies in the present work

Type of sample	Sample Characteristics	Total amount of PS2 RC (in terms of Chl equivalent)	Factor (fold PS2 RC)
Leaves (100 mg leaves in rotor)	PS1, PS2 RC and associated core antenna and light harvesting antenna	1 ng (nanogram)	1x
Thylakoid (~140 µg Chl/70 µl of sample)	PS1, PS2 RC and associated core antenna and light harvesting antenna	2 ng	2x
BBY (~200 µg Chl/70 µl of sample)	PS2 RC associated with core antenna (CP43, CP47) and LH2 antenna	4 µg (microgram)	$4x10^4$
PS2 core	PS2 RC associated with core antenna (CP43, CP47)	40 µg	$4x10^5$
D1D2	PS2 RC (pure D1D2 cytochrome b-55)	200 µg	$2x10^6$

REFERENCES

1. Polenova, T. & McDermott, A. E. A coherent mixing mechanism explains the photoinduced nuclear polarization in photosynthetic reaction centers. *J. Phys. Chem. B* **103**, 535-548 (1999).

2. Egorova-Zachernyuk, T. A., van Rossum, B., Boender, G.- J., Franken, E., Ashurst, J., Raap, J., Gast, P., Hoff, A. J., Oschkinat, H. & de Groot, H. J. M. Characterization of Pheophytin Ground states in *Rhodobacter sphaeroides* R26 Photosynthetic Reaction Centers from Multispin Pheophytin Enrichment and 2-D ^{13}C MAS NMR Dipolar Correlation Spectroscopy. *Biochemistry* **36**, 7513-7519 (1997).

3. Boender, G. J., Raap, J., Prytulla, S., Oschkinat, H. & de Groot, H. J. M. MAS NMR structure refinement of uniformly ^{13}C enriched Chl a / water aggregates with 2D dipolar correlation spectroscopy. *Chem. Phys. Lett.* **237**, 502-508 (1995).

4. Sai Sankar Gupta, K. B., Alia, A., de Groot, H. J. M. & Matysik, J. Symmetry break of special pair: Photochemically induced dynamic nuclear polarization NMR confirms control by non-aromatic substituents. *J. Am. Chem. Soc.* **135**, 10382-10387 (2013).

5. Sai Sankar Gupta, K. B., Daviso, E., Jeschke, G., Alia, A., Ernst, M. & Matysik, J. Spectral-editing through laser-flash excitation in two-dimensional photo-CINDP MAS NMR experiments. *J. Magn. Reson.* **246**, 9-17 (2014).

6. Paul, S., Bode, B., Matysik, J. & Alia, A. Photochemically induced dynamic nuclear polarization observed by solid-state NMR in a uniformly 13C-isotope labeled photosynthetic reaction center. *J. Phys. Chem. B* **119**, 13987-13903 (2015).

7. Daviso, E., Alia, A., Prakash, S., Diller, A., Gast, P., Lugtenburg, J., Matysik, J. & Jeschke, G. Electron-nuclear spin dynamics in a bacterial reaction center. *J. Phys. Chem. C* **113**, 10269-10278 (2009).

8. Jeschke, G. & Matysik, J. A reassessment of the origin of photochemically induced dynamic nuclear polarization effects in solids. *Chem. Phys.* **294**, 239-255 (2003).

9. Tamarath, S. S., Bode, B. E., Prakash, S., Sai Sankar Gupta, K. B., Alia, A., Jeshke, G. & Matysik, J. Electron spin density distribution in the special pair triplet of Rhodobacter sphaeroides R26 revealed by magnetic field dependence of the solid-state photo-CIDNP effect. *J. Am. Chem. Soc.* **134**, 5921-5930 (2012).

10. Tamarath, S. S., Alia, A., Daviso, E., Mance, D., Golbeck, J. H. & Matysik, J. Whole-cell NMR characterization of two photochemically active states of the photosynthetic reaction center in heliobacteria. *Biochemistry* **51**, 5763-5773 (2012).
